# Ectodermal dysplasia (ED) syndrome

**DOI:** 10.7603/s40681-014-0027-9

**Published:** 2014-11-26

**Authors:** Siew-Yin Chee, Chung-Hsing Wanga, Wei-De Lina, Fuu-Jen Tsaia

**Affiliations:** 1 College of Medicine, China Medical University, 404 Taichung, Taiwan; 2 Department of Pediatrics and Medical Genetics, Children’s Hospital, China Medical University Hospital, 404 Taichung, Taiwan; 3 Department of Medical Research, China Medical University Hospital, 404 Taichung, Taiwan

**Keywords:** Ectodermal dysplasia (ED)

## Abstract

Ectodermal dysplasia (ED) syndrome comprises a large, heterogeneous group of inherited disorders that are defined by primary defects in the development of 2 or more tissues derived from the embryonic ectoderm. The tissues primarily involved are the skin and its appendages (including hair follicles, eccrine glands, sebaceous glands, nails) and teeth. The clinical features include sparse hair, abnormal or missing teeth, and an inability to sweat due to lack of sweat glands. One such case report of ectodermal dysplasia is presented here.

## Introduction

Ectodermal dysplasia (ED) syndrome is a rare heterogeneous group of inherited disorders that share primary defects in the development of two or more tissues derived from the ectoderm. These tissues primarily affected are the skin, hair, nails, eccrine glands, and teeth. Defects in tissues derived from other embryologic layers are not uncommon. The disorders are congenital, diffuse, and nonprogressive. To date, more than 192 distinctive syndromes have been described with all possible modes of inheritance. The most common syndromes within this group are hypohidrotic (anhidrotic) ED and hidrotic ED. Hypohidrotic ED (also known as Christ-Siemens-Touraine syndrome) is the more common phenotype and is usually inherited as an X-linked recessive trait. It is characterized by several defects (e.g. hypohidrosis, anomalous dentition, onychodysplasia, hypotrichosis). Typical facies are characterized by frontal bossing, sunken cheeks, a saddle nose, thick and everted lips, wrinkled and hyperpigmented skin around the eyes, and large, low-set ears. Dental manifestations include conical or pegged teeth, hypodontia or complete anodontia, and delayed eruption of permanent teeth. Eccrine sweat glands may be absent or sparse and rudimentary, particularly in those with hypohidrotic ED. In some cases, mucous glands are absent in the upper respiratory tract and in the bronchi, esophagus, and duodenum. Scalp hair may be sparse, short, fine, dry, or there may be a complete absence of hair. Structural hair-shaft abnormalities like longitudinal grooving, hair-shaft torsion, and cuticle ruffling can also be seen. The prevalence of atopic eczema is high. Other common signs are short stature, eye abnormalities, decreased tearing, and photophobia. Intelligence in those affected is normal. Nails are often brittle and thin or show abnormal ridging, but they may be grossly deformed especially in the hidrotic type. The presence or absence of these abnormalities defines the different types of this syndrome.

## Case report

**Fig. 1:**
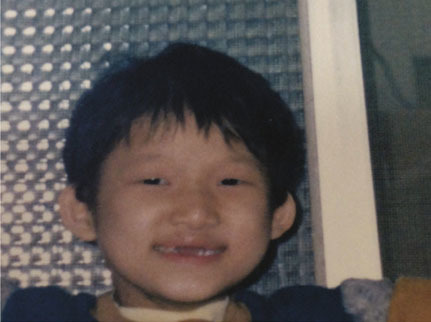
The patient was 5 year-old with obvious cone-shape teeth and scanty eyebrows.

**Fig. 2:**
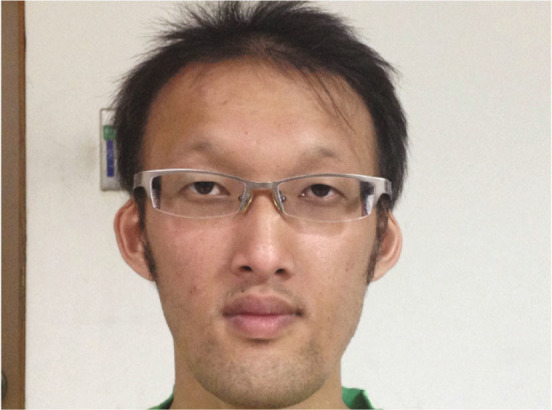
Frontal bossing, prominent supraorbital ridge, sunken cheeks, thick lips, low-set ears, scanty eyebrows and hypotrichosis with fine, sparse and brittle scalp hair but normal sexual hair (beard).

**Fig. 3:**
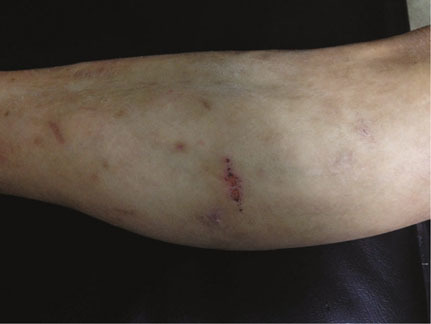
Fragile-appearing dry skin with reduced hair follicles

A 27-year-old man was presented to the outpatient department with hypohidrosis and scant body hair. He had a past medical history of asthma with frequent attacks and regularly inhaled corticosteroid and bronchodilator. Easy hyperthermia with hypohidrosis was noted during childhood. He also had malformed teeth all with a cone-shaped aspect (Figure 1), but he received teeth replacements 10 years ago. The clinical phenotype of the patient was characterized by frontal bossing, a prominent supraorbital ridge, sunken cheeks, thick lips, sparse hair, scanty eyebrows, and low-set and overfolding ears (Figure 2). A physical examination showed hypotrichosis with fine, sparse and brittle scalp hair with excessive fragility (Figure 3). Body hair was also diminished and sweat glands were found only over the axilla region. Sexual hair, beard and pubic hair, was normal. The patient also had generalized dry and hypopigmented skin with reduced hair follicles. Dermatitis resembling atopic skin was mainly noted over his bilateral extremities. None of his family member had similar symptoms. Hypohidrotic ED was impressed.

## Discussion

ED syndrome is a group of genetic disorders identified by lack or dysgenesis of at least two ectodermal derivatives such as hair, nails, teeth, or sweat glands. Obvious manifestations of the disorders are not clinically apparent in newborns. They normally become evident during infancy or early childhood. The patient reported here had involvement of hair, sweat glands, and teeth. Other ectodermal structures were largely unaffected. In addition, he had atopic eczema, frontal bossing, sunken cheeks and low set ears. These clinical features were supportive in diagnosing hypohidrotic ED. Currently, 64 genes and 3 chromosomal loci [1] have been identified in about 62 different ED syndromes. Hypohidrotic ED is the most frequent form of ED syndrome that can be inherited in an X-linked (XL), autosomal recessive (AR) or (AD) autosomal dominant manner. The XL recessive type is the most common form of hypohidrotic ED. 94% of the patients carries the mutation of the *EDA1* gene. This defective gene was cloned thereby leading to the identification of a novel signaling molecule of the tumor necrosis factor (TNF) superfamily named ectodysplasin (EDA) [2]. Autosomal forms of hypohidrotic ED are due to mutations in the EDA receptor (EDAR), a novel TNF receptor family member. Currently over 100 different mutations in the EDA gene have been reported, while only 20 causative mutations are known for EDAR. Morbidity and mortality are related to the absence or presence of eccrine and mucous glands. Children with decreased sweating may have a mortality rate of up to 30% in infancy or early childhood because of intermittent hyperpyrexia. No definite pharmacological treatment is available, and the management of affected patients depends on which structures are involved. Patients with hypohidrotic ED are advised to wear light clothing, to carry a cold-water spray bottle, and to stay in air conditioned environments whenever possible. For patients with dental defects, early dental evaluation and intervention is advised, as is routine dental hygiene. Orthodontic treatment may be undertaken for cosmetic reasons and to ensure adequate nutritional intake. Life expectancy in such cases where the necessary precautions are observed, is almost as good as in other, non-affected children.

## Conclusion

Ectodermal dysplasia syndrome is a rare genetic disorder with the involvement of various tissues in the body. A careful and a thorough examination of a patient will lead to an accurate diagnosis. It should be noted that an absence of a positive family history for ectodermal dysplasia should not be a factor in causing any diagnostic dilemmas with respect to ectodermal dysplasia, a condition that shows multiple modes of inheritance.
